# Marginal adaptation of 3D-printed crowns: influence of preparation geometry and digitization workflow

**DOI:** 10.1186/s12903-026-09649-w

**Published:** 2026-08-27

**Authors:** Khalid Abdelsalam, Ahmed Attia

**Affiliations:** 1 Dept of Fixed Prosthodontics, Faculty of Dentistry, Mansoura University, Mansoura, Egypt; 2 Dept. of Fixed Prosthodontics, Faculty of Dentistry, Mansoura University, El Gomhouria St, Mansoura, Dakahlia Governorate Egypt

**Keywords:** 3D printing, Additive manufacturing, Resin matrix ceramic, Marginal gap, Finish line width, Artificial aging

## Abstract

**Background:**

This in vitro study evaluated the impact of different workflow and finish line design on the marginal adaptation of 3D printed resin matrix ceramic crowns.

**Methods:**

Forty extracted mandibular molars were randomly allocated into two groups according to finish line design (*n* = 20); heavy chamfer 0.8 mm (H) and conservative chamfer 0.2 mm (C). Each main group was divided into 2 subgroups (*n* = 10) according to workflow; direct digital intraoral scanning workflow (D) and semi-digital workflow (S). Crowns were 3D printed from hybrid resin and cemented using adhesive resin cement. Marginal adaptation was evaluated for all groups using a stereomicroscope before cementation and after the combined cementation and artificial aging. Statistical analysis was performed with 3-way ANOVAs and post hoc Tukey test, (*P* < 0.05).

**Results:**

Significant effects were found for finish line design, workflow, and the combined cementation and artificial aging (*P* < 0.05), with no significant interaction between finish line design and workflow (*P* = 0.552). Mean marginal gaps (µm) of test groups before cementation and after the combined cementation and aging protocol were: HD, (71.72 ± 4.82), (84.5 ± 2.75); CD, (82.16 ± 3.76), (97.32 ± 3.16); HS, (92.51 ± 3.64), (111.8 ± 3.37); and CS, (106.1 ± 3.57), (123.4 ± 2.58). All groups showed significant increases thereafter (*P* < 0.05). Heavy chamfer finish line and direct digital workflow each yielded significantly smaller marginal gap than conservative chamfer and semi-digital workflow at both time points (*P* < 0.05).

**Conclusions:**

Within the limitations of this in vitro study, finish line design and workflow type each independently influenced the marginal adaptation of 3D-printed resin matrix ceramic crowns. A heavy chamfer and a direct digital workflow were each associated with smaller marginal gaps.

## Background

The incorporation of digital technologies in dentistry has considerably improved the accuracy and efficacy of dental restorations plus chairside fabrication of restorations [1–4]. The use of additive manufacturing technologies employing three-dimensional (3D) printing increased as a modern manufacturing technique alternative to subtractive CAD-CAM systems [5, 6]. Data acquisition of prepared teeth can be obtained either by direct digitization from the abutment using an intraoral scanner or through indirect digitization of a conventional definitive cast utilizing an extraoral scanner followed by fabrication of the restoration [7–11]. Accuracy in these two processes culminates in the ultimate adjustment of the restorations [5, 12–14]. The indirect approach is disadvantageous due to its reliance on a conventional impression and working stone cast with possible dimensional changes [5, 13].

Marginal adaptation is considered one of the most crucial factors influencing the long-term clinical success of permanent tooth-supported restorations. A marginal gap of up to 120 μm has been determined as the clinically acceptable limit [15]. Gaps over this threshold predispose restorations to cement disintegration, microleakage, and secondary caries [16]. The marginal fit is affected by several factors, including the impression workflow for capturing the prepared tooth geometry, the design of the finish line in the tooth preparation, the fabrication technology employed, and the luting protocol utilized [16–19]. Literature consistently reports an inevitable increase in marginal gap with the cementation of definitive restorations, primarily due to the hydraulic pressure and film thickness of the cement, rendering both pre-cementation marginal fit and post-cementation marginal gap critical clinical outcome parameters [16, 20, 21].

The impression workflow constitutes a principal cause of dimensional inaccuracies in crown fabrication. Conventional polyvinyl siloxane impressions, although dimensionally stable, require multiple laboratory steps that introduce cumulative dimensional error [22–24]. In contrast, intraoral digital scanners precisely capture the prepared tooth’s geometry in one digital workflow, shortening the process of restoration fabrication. Meta-analyses and systematic reviews show that intraoral scanning provides a marginal fit for single-unit restorations that is comparable or better than conventional impression techniques [25, 26]. However, most studies comparing these two workflows have concentrated on milled ceramic or zirconia restorations; evidence explicitly investigating the impact of impression workflow on 3D-printed definitive resin crowns is still scarce [27, 28], representing a clinically relevant gap in the current literature.

The design of the finish line is another crucial factor influencing marginal adaptation and the structural integrity of crowns [29, 30]. The chamfer finish line is frequently recommended because of its technical simplicity and conservative tooth reduction; however, finish line width can considerably affect scanner capture quality and the capability of CAD software to accurately identify the preparation border [18]. Conventional chamfer preparations are generally recommended at 0.3–0.5 mm [31], whereas evidence maps for all-ceramic CAD/CAM posterior crowns have proposed at least 1.0 mm [19]. Therefore, the 0.2-mm configuration was not selected as a conventional clinical reference, but as a deliberate challenge condition. This width approaches the lower limit of reliable optical capture. Finish line distinctness and accuracy vary substantially between scanning systems even when their overall accuracy is comparable, indicating that margin capture is governed by localized resolution at the finish line rather than by general scanner trueness [32]; narrow and shallow preparation geometries correspondingly yield the largest scanning deviations, attributed to restricted optical access and reduced light reflection [33]. This design was intended to assess how a more conservative finish line, compared with a 0.8-mm heavy chamfer, may influence margin detection and marginal adaptation in different workflows, while also remaining tooth-conserving and avoiding the limitations associated with vertical preparations lacking a defined finish line reported previously [34].

Notwithstanding these findings, the comparative impact of heavy versus conservative chamfer finish line designs on the marginal adaptation of three-dimensional (3D) printed resin crowns has not been explicitly investigated [28]. The objective of this study was to assess the influence of various workflows and finish line designs on the marginal adaptation of 3D printed resin matrix ceramic crowns. The null hypothesis was that workflow type, finish line design, and combined cementation and artificial aging would have no influence on the marginal adaptation of 3D-printed resin matrix ceramic crowns.

## Materials and methods

### Materials

The materials utilized in this study are listed in (Table 1).

**Table 1:** Materials used in the study in detail

Product name	specification	composition	Manufacturer	Lot number
VarseoSmile TriniQ	ceramic-filled hybrid 3D printing resin	4,4′ -isopropylidenediphenol, ethoxylated and 2-methylprop-2-enoic acid, Benzene acetic acid, alpha–oxo- methyl ester, diphenyl (2,4,6-trimethylbenzoyl) phosphine oxide. (Exact composition not provided by the company.)	BEGO Bremer Bremen, Germany	602,211
Monobond N	Universal primer	Alcohol solution of silane methacrylate, phosphoric acid methacrylate, and sulphide methacrylate	Ivoclar Vivadent, Schaan, Liechtenstein	Z066MZ
Multilink N primer A	Self-curing and self-etching primer	an aqueous solution of initiators.	Ivoclar Vivadent	Z09MZP
Multilink N primer B	Self-curing and self-etching primer	HEMA, phosphonic acid and methacrylate monomers.	Ivoclar Vivadent	Z09LZK
Multilink N	Dual cure resin cement	The monomer matrix is composed of dimethacrylate and HEMA. The inorganic filler includes barium glass, ytterbium trifluoride, and spheroid mixed oxide. Particle size is 0.25–3.0 μm. Total inorganic filler volume is 40%	Ivoclar Vivadent	Z09G11

Sample size was calculated based on the marginal gap values in a previous study [35] using G*Power (version 3.1.9.7; University of Düsseldorf, Düsseldorf, Germany) [36]. An a priori power analysis was performed for a one-way fixed-effects ANOVA. Based on an effect size (f) of 1.1, a significance level (α) of 0.01, a statistical power (1 − β) of 99%, and 4 study subgroups, the calculated total sample size was determined to be 32 specimens (8 specimens per subgroup), so the total sample size was increased to 40 specimens (10 specimens per subgroup) to compensate for pre-test failures. This calculation achieved an actual statistical power of 0.992.

The protocol of this study was approved by the ethical committee of the Faculty of Dentistry, Mansoura University with approval number. (M01012024FP). Forty intact human mandibular molars of uniform size and morphology, and free of caries, cracks lines and fractures, recently extracted for periodontal reasons, were collected. Following the removal of soft tissue using hand scalers, the teeth were preserved at 4 °C in a 0.5% saturated thymol solution in distilled water to inhibit microbial proliferation [37].

To facilitate handling during preparation, impression, and cementation procedures, the roots were coated with molten wax up to the marked level (2 mm apical to the cemento-enamel junction (CEJ) to simulate the periodontal ligament space (0.20–0.30 mm) [38]. An auto polymerizing acrylic resin was mixed and poured into Teflon molds then the roots of each tooth were vertically embedded in acrylic resin blocks. After polymerization, teeth were removed, creating alveolus-shaped sockets. Residual wax was eliminated. Both roots and sockets were coated with tray adhesive (Zhermack, Badia Polesine RO, Italy). Polyvinyl siloxane light-body material (Elite HD+; Zhermack) was injected, and teeth were repositioned. Excess material was removed. The finish line was established 1 mm coronal to the CEJ.

### Molar preparation

A silicone index (Zetaplus; Zhermack) was fabricated and sectioned buccolingually with a #15 blade to evaluate occlusal and axial reduction. Teeth were randomly assigned into two equal groups (*n* = 20). Group (H), teeth were prepared with a heavy chamfer finish line 0.8 mm. Group (C), teeth were prepared with conservative chamfer finish line 0.2 mm. All tooth preparations were performed by a single, experienced operator. The occlusal preparation was standardized by creating occlusal guiding grooves with a depth marker set 1 mm (Microscopy, USA) to achieve a 1 mm reduction, while adhering to the occlusal anatomy. Axial tooth reductions were performed while mounting the teeth on a dental surveyor (Saeshin surveyor II, Saeshin Co, Korea) attached to a straight handpiece to maintain a consistent path of insertion and ensure a total convergence angle of 6°. All preparations maintained identical occlusal reduction of 1 mm and axial convergence angle of 6 degrees. Finishing and polishing were performed with fine and ultra-fine diamond instruments under copious water cooling.

Each group was subsequently subdivided into two subgroups (*n* = 10) according to workflow; direct digital intraoral scanning workflow (D), in which impressions were obtained directly using an intraoral scanner (Medit i700, MEDIT Corp, Seoul, Korea), and semi-digital workflow (S), in which impressions were made using a two-step putty-wash technique with addition silicone (Elite HD+; Zhermack), followed by pouring Type IV stone casts. The casts were then digitized using an extraoral laboratory scanner (Medit T310).

### Crown fabrication

Crowns were designed using Dental DB 2.2 Valletta software (Exocad GmbH) and nested in software (AccuWare; SHINING 3D, Hangzhou, China) for support generation and orientation. Prior to restoration design, finish line width was verified digitally on the scans of all prepared teeth using the 2D cross-sectional view and 3D distance measurement tools of the same software. During design, the margin detection tool was used to delineate the preparation finish line, while the cross-sectional view and digital measurement tools confirmed restoration thickness.

Crowns were fabricated from ceramic-filled hybrid 3D printing resin (VarseoSmile TRIniq) according to the recommended printing protocol by the manufacturer using processing system (AccuFab-CEL; SHINING 3D). Post-processing included cleaning in isopropyl alcohol (FabWash; SHINING 3D) for 3 min followed by air-drying and UV post-curing unit (FabCure; SHINING 3D). Finally, supports were removed and crowns were finished and polished. Following the 3D-printing process, the thickness of each restoration was measured and confirmed using a calibrated digital caliper.

### Pre-cementation marginal gap evaluation

**Fig. 1:**
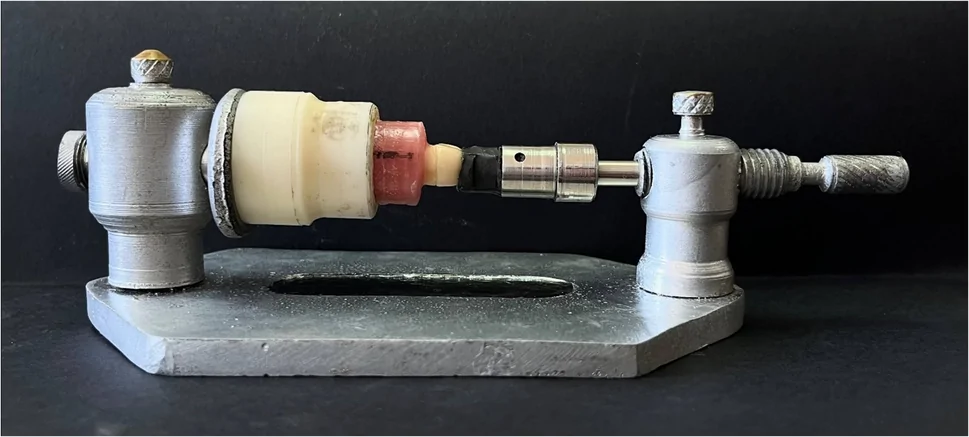
Showing the sample positioning device (SPD)

**Fig. 2:**
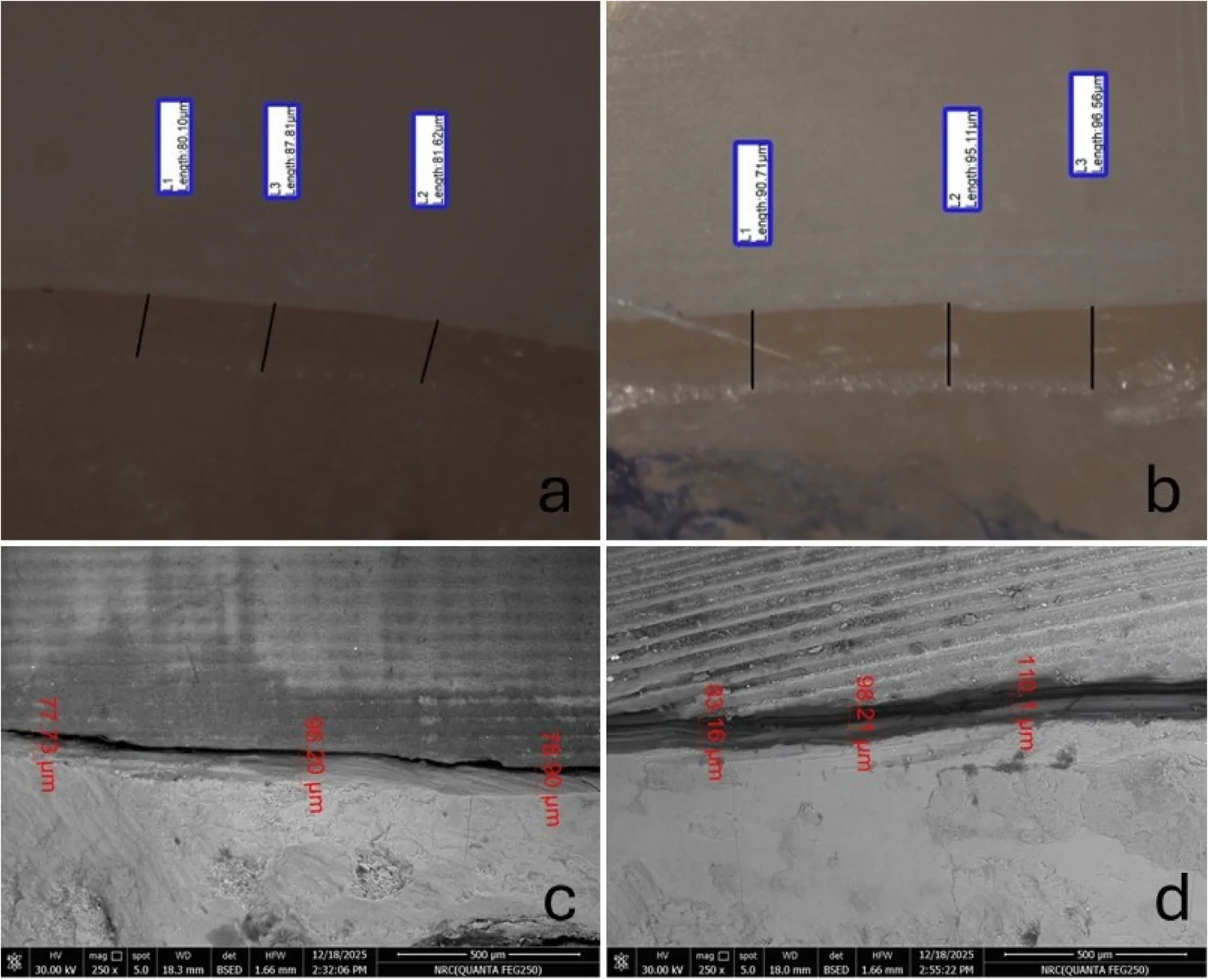
Showing marginal gap before cementation. **a**, **b** Screen shot of stereomicroscope. **c**, **d** Representative SEM micrographs, (Original magnification, ×250)

All crowns were seated on their corresponding prepared teeth and were stabilized using specially fabricated device [39]. Also, specimen orientation and measurement angles were standardized relative to the stereomicroscope using a custom Sample Positioning Device (SPD). The SPD comprises a stationary base, reference indicators, a calibrated gauge, and a coil spring that applies a constant seating force. Each specimen was mounted within the rotatable housing by aligning pre-marked reference points. The housing containing the sample can rotate around the stationary base and can be secured at each of the 12 positions for accurate measurement (Fig. 1). Vertical marginal gap was evaluated using a stereomicroscope (SZ2-ILST; Olympus Corp, Tokyo, Japan) at 40× magnification with a digital camera (Tucsen Photonics). A total of 12 measurements per specimen were recorded (three points per surface) to ensure reliability [39] (Fig. 2a and b). For each specimen, the 12 circumferential marginal gap measurements were averaged into a single mean value per evaluation time point. All measurements were conducted by a single operator who was blinded to group allocations. Before formal data collection, examiner calibration was established using pilot specimens not included in the study. To verify the marginal gap measurements obtained by stereomicroscope, one representative specimen from each group was evaluated using a QUANTA FEG 250 field emission scanning electron microscope (FESEM) (Thermo Fisher Scientific, Waltham, MA, USA). SEM was employed as a supplementary confirmatory method to assess marginal gap values. A total of 12 measurements per specimen (three points per surface) were taken directly using the integrated SEM measurement software after being calibrated in accordance with the microscope scale bar (500 μm) and obtained from several predetermined points along the tooth-restoration interface in perpendicular orientation to ensure reproducibility and standardization. The SEM mean values for the representative specimens were consistent with the stereomicroscope findings (Fig. 2c and d).

### Cementation of the restorations

The intaglio surfaces of the crowns were air-abraded with 50 μm Al_2_O₃ particles using a sandblaster (Renfert Basic Classic) at 2 bar (0.2 MPa) for 10 s at a 10 mm distance [40]. Crowns were ultrasonically cleaned in 99% isopropanol for 3 min and air-dried [26]. A universal primer (Monobond N) was applied for 60 s and air-dried. Prepared teeth were cleaned and dried, and primer A & B was applied and scrubbed for 30 s followed by thinning with air. Dual cure adhesive resin cement (Multilink N) was auto mixed and applied directly onto the intaglio surface of each crown. Crowns were kept under constant load using a 5-kg load device during cementation. Initial light curing was performed for 2 s per surface, excess cement was removed and glycerin was applied to prevent oxygen inhibition. Final polymerization was completed with 20 s curing per margin. Finishing and polishing was performed using a diamond polishing system (Zircovis Polishing Kit; Kenda AG, Vaduz, FL) to obtain optimal surface smoothness and luster.

### Artificial aging

After cementation, all specimens were submerged in a water bath at 37 °C for 6 months. Long-term water storage at 37 °C is an established method for inducing hydrolytic degradation of resin-based materials and of the adhesive interface [41]. Specimens were thermal cycled for 10,000 cycles between 5 °C and 55 °C, with a dwell period of 30 s for each cycle [6] (Thermo Scientific, Thermo Fisher Scientific Inc., Waltham, USA). Furthermore, the specimens were subjected to cyclic loading for 120,000 cycles using masticatory simulator (Willitec Kausimulator, Willitec, Munich, Germany) under wet conditions to mimic one year clinically [42]. Steatite ceramic balls (4 mm diameter; Hoechst Ceram Tec, Wunsiedel, Germany) were employed as antagonistic surfaces to replicate the opposing teeth. The loading cycle frequency was 1.2 Hz, with a kinetic energy of 2,250 × 10^–6^ J, a maximum load of 49 N, and a minimum load of 0 N [43, 44].

### Marginal gap evaluation after cementation and artificial aging

**Fig. 3:**
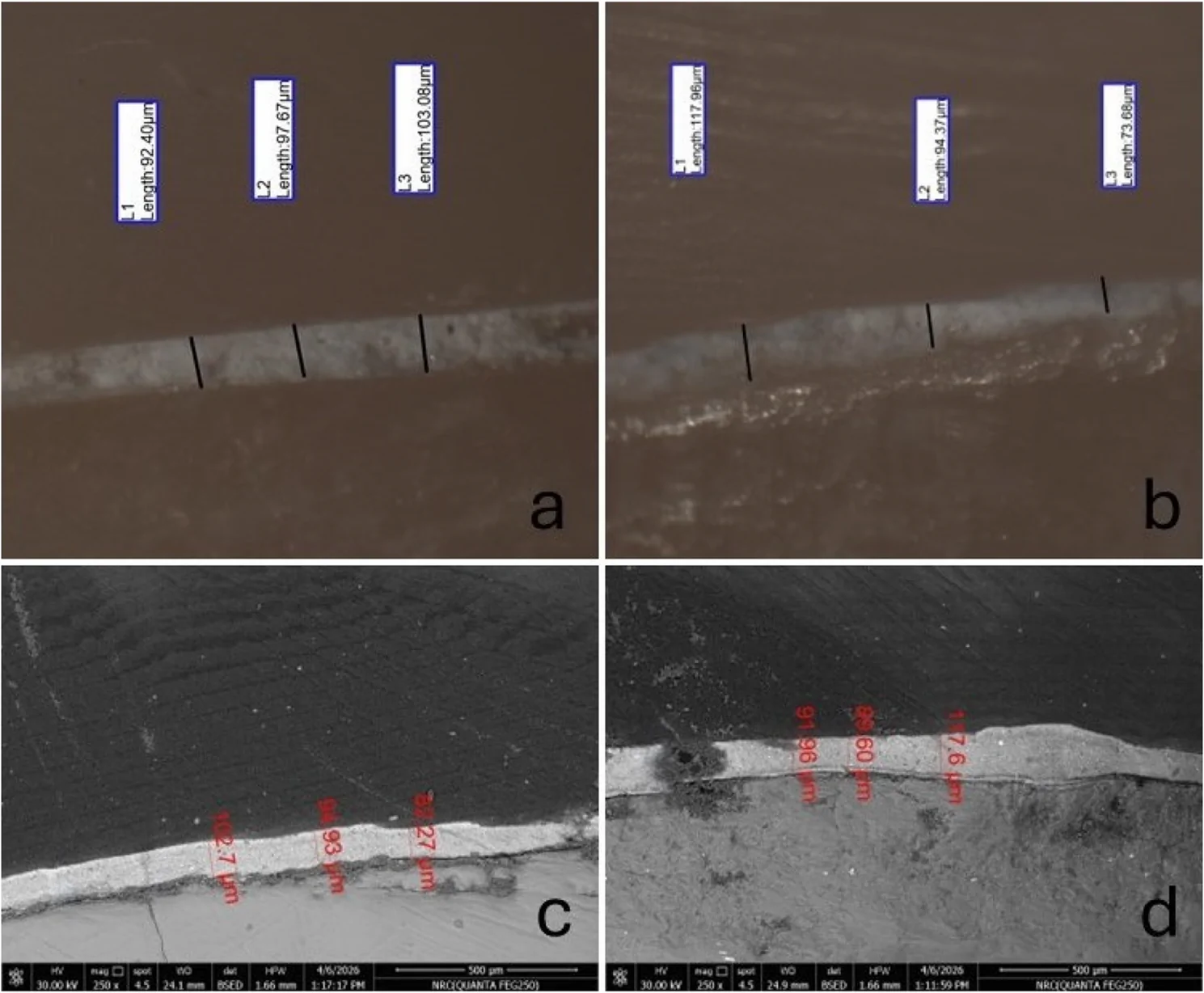
Showing marginal gap after the combined cementation and artificial aging. **a**, **b** Screen shot of stereomicroscope. **c**, **d** Representative SEM micrographs, (Original magnification, ×250)

All specimens were intact after cementation and artificial aging process [2]. Marginal gap was assessed again for all specimens as mentioned before cementation [39] (Fig. 3a and b). Also, the same four samples were evaluated by SEM (Fig. 3c and d). Intra-examiner reliability was assessed by re-measuring 10 randomly selected specimens (25% of the sample), after an interval of two months, with each measurement repeated at the same predetermined sites.

## Results

Statistical software (SPSS version 27 SPSS, Inc.) was used for statistical analyses. Data were normally distributed (Shapiro-Wilk test, *P* > 0.05). Therefore, parametric tests were utilized for statistical analysis. A single full-factorial three-way ANOVA was used to evaluate the main and interaction effects of the three independent variables (Table 2). Post Hoc Tukey-honest significant difference (HSD) test at (*P* < 0.05) was applied for multiple comparisons (Table 3). For each specimen, the 12 circumferential marginal gap measurements were averaged into a single mean value per evaluation time point, which served as the unit of statistical analysis.

**Table 2:** Three-way ANOVA for the effects of finish line design, workflow type, cementation and artificial aging stage on marginal gap

By level	Sum of squares	Df	Mean square	F	*P*-values
Corrected model	20288.656a	7	2898.379	234.672	< 0.001*
Intercept	740201.288	1	740201.288	59931.553	< 0.001*
Finish line design	2928.200	1	2928.200	237.086	< 0.001*
Type of workflow	12034.418	1	12034.418	974.385	< 0.001*
Combined cementation & artificial aging	5203.538	1	5203.538	421.313	< 0.001*
Finish line design * type of workflow	4.418	1	4.418	0.358	0.552
Finish line design * Cementation & artificial aging	0.128	1	0.128	0.010	0.919
Type of workflow * Cementation & artificial aging	93.312	1	93.312	7.555	0.008*
Finish line design * type of workflow * Cementation & artificial aging	24.642	1	24.642	1.995	0.162
Error	889.256	72	12.351		
Total	761379.200	80			
Corrected Total	21177.912	79			

(*) Indicate statistically significant difference

**Table 3:** Showing means ± SD marginal fit in (µm) of test groups and results of Post Hoc Tukey (HSD) test at (*P* < 0.05)

Time			Before cementation				After combined cementation and artificial aging			
		Mean ± SD	HD	HS	CD	CS	HD	HS	CD	CS
Before cementation	HD	71.72 ± 4.82		< 0.001*	< 0.001*	< 0.001*	< 0.001*	< 0.001*	< 0.001*	< 0.001*
	HS	92.51 ± 3.64			< 0.001*	< 0.001*	< 0.001*	< 0.001*	0.06	< 0.001*
	CD	82.16 ± 3.76				< 0.001*	0.811	< 0.001*	< 0.001*	< 0.001*
	CS	106.10 ± 3.57					< 0.001*	0.01*	< 0.001*	< 0.001*
After combined cementation and artificial aging	HD	84.50 ± 2.75						< 0.001*	< 0.001*	< 0.001*
	HS	111.80 ± 3.37							< 0.001*	< 0.001*
	CD	97.32 ± 3.16								< 0.001*
	CS	123.40 ± 2.58								

*HD* Heavy chamfer/ Direct digital intraoral scanning workflow, *HS* Heavy chamfer/ Semi-digital workflow, *CD* Conservative chamfer/ Direct digital intraoral scanning workflow, *CS* Conservative chamfer/ Semi-digital workflow

(*) Indicate statistically significant difference

Three-way ANOVA revealed that all three independent variables including; finish line design, workflow type, the combined cementation and artificial aging had a statistically significant effect on the marginal gap (*P* < 0.001). Most interaction effects were not significant (*P* > 0.05) except for workflow type × combined cementation and artificial aging (*P* = 0.008), indicating that the increase in marginal gap differed depending on the workflow used.

**Fig. 4:**
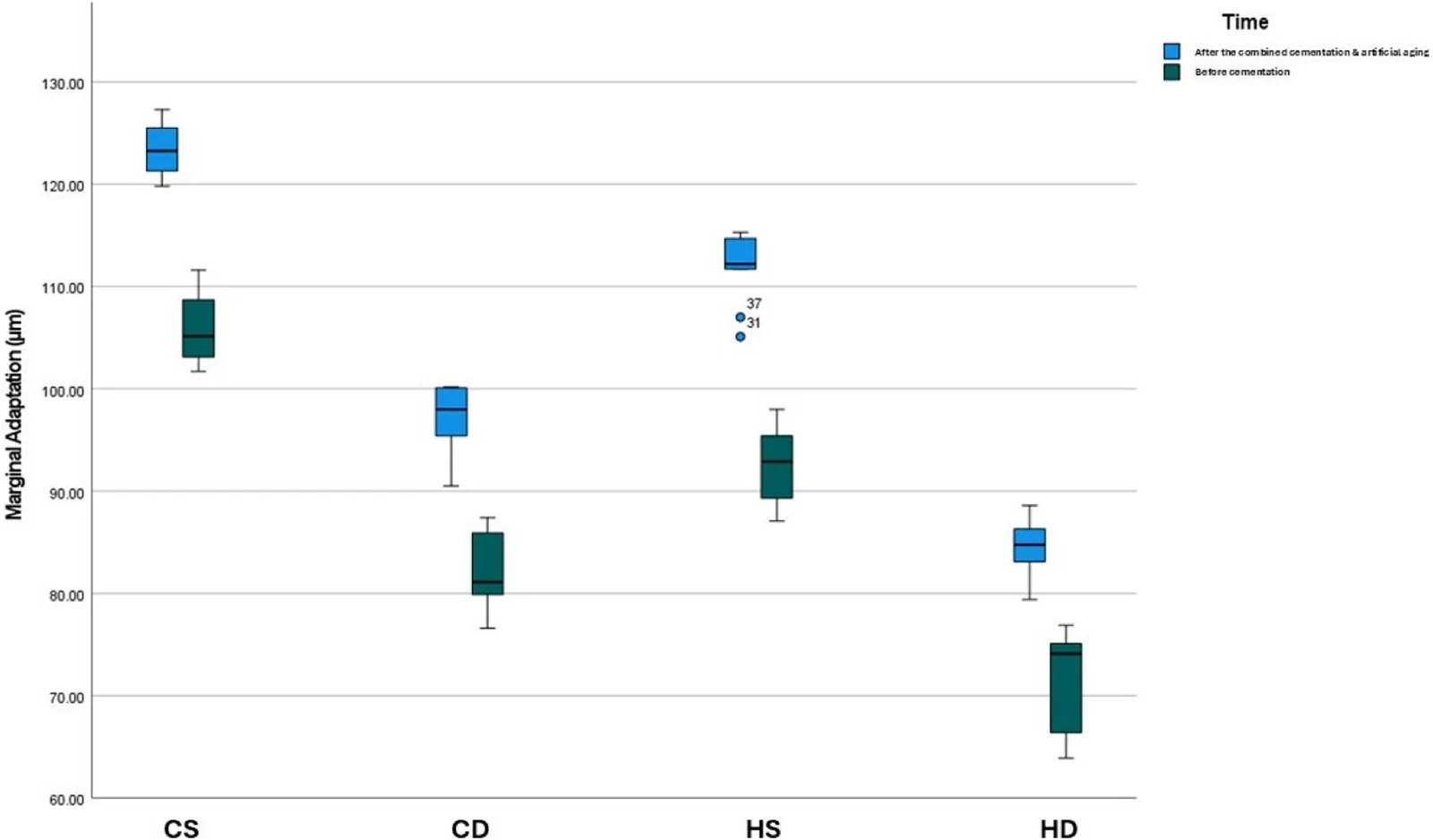
Box plots representing mean marginal adaptation for all test groups before cementation and after the combined cementation and artificial aging

Post Hoc Tukey (HSD) test (Table 3) showed a considerable increase in marginal gap in all test groups after the combined cementation and artificial aging, with an average increase from 12.78 to 19.28 μm. The HD group showed the lowest overall marginal gap 71.72 ± 4.82 μm before cementation and 84.50 ± 2.75 μm after the combined cementation and artificial aging, (*P* < 0.001) whereas the CS group had the highest values 106.10 ± 3.57 μm and 123.40 ± 2.58 μm, respectively (*P* < 0.001). Test groups (HD&CD) had markedly reduced marginal gaps in comparison to test groups (HS&CS) (*P* < 0.001), and heavy chamfer (0.8 mm) demonstrated better marginal adaptation than the conservative chamfer (0.2 mm) at both time points (*P* < 0.001). Results were further illustrated by Box plots (Fig. 4). The marginal gap analysis yielded an Intraclass Correlation Coefficient of 0.986, reflecting excellent reliability.

## Discussion

Based on the findings of the present investigation, the null hypothesis was rejected. Statistically significant differences in marginal gap values were found across the four test groups before cementation and after the combined cementation and artificial aging. The finish line design, workflow type and the combined cementation and artificial aging had a statistically significant effect on marginal adaptation outcomes. Regarding the workflow, intraoral scanning was associated with significantly lower marginal gap values than the conventional polyvinyl siloxane impression followed by extraoral scanning for both finish line designs. The inferior marginal adaptation observed in the extraoral scanner test groups might be attributed to the cumulative nature of errors introduced across the multi-step semi digital workflow. While polyvinyl siloxane addition silicone materials used in this study, are among the most dimensionally stable impression materials available with minimal dimensional change and absence of polymerization by-products [24], The semi-digital workflow still involves impression removal and deformation recovery, transportation, model pouring, and subsequent extraoral scanning, each adding incremental dimensional distortions [22]. In contrast, intraoral scanning removes these intermediate phases, immediately recording the preparation geometry in a singular digital procedure, which may reduce the likelihood of cumulative error. This finding agreed with other studies which demonstrated that crowns produced by intraoral scanning were better in terms of marginal adaptation than those fabricated from conventional silicone impressions [25, 26].

Considering finish line design, the heavy chamfer configuration was associated with a smaller marginal gap for the 3D-printed resin matrix ceramic crowns evaluated in this study, consistent with prior reports on CAD/CAM-milled restorations, in which the effect was attributed to the increased material bulk at the cervical margin and the more distinctly defined preparation boundary it presents to both scanner optics and CAD software [29]. The broader heavy chamfer finish line (0.8 mm) facilitates unambiguous angular transition at the margin, which may be captured more reliably by intraoral scanning technology and identified more consistently by the margin-detection algorithms of design software [18]. In contrast, conservative chamfer (0.2 mm), offers conservative tooth reduction, a more subtle and shallower angular transition that is inherently more challenging to resolve accurately, which may increase the risk of margin misidentification during the virtual design phase and could partly explain the larger marginal gap observed for this group [18]. These findings were consistent with the established evidence that finish line width and geometry significantly influence the marginal adaptation of CAD/CAM-fabricated restorations [18, 19]. All pre-cementation marginal gap values recorded in the present study ranging from 71.72 μm in the heavy chamfer direct digital group (HD) to 106.1 μm in the conservative chamfer semi-digital group (CS) still within the clinically acceptable marginal gap limit of 120 μm as reported previously [15]. After cementation and artificial aging, marginal gap values ranged from 84.50 μm (HD) to 123.4 μm (CS), a modest but statistically significant increase of approximately 10 to 20 μm across all groups. Although the CS group marginally exceeded the clinically acceptable 120 μm limit at the second time point, this increment fell within the range widely reported after cementation [20]. This increase in marginal gap is likely attributable to the combined effects of cementation and artificial aging: during crown seating, the hydraulic pressure generated by the viscous luting agent, combined with its inherent film thickness, physically elevates the restoration, resulting in an increase of the marginal gap [16, 28]. The aging regimen that followed was designed to approximately simulate 2.5 years of clinical service, based on the cumulative effect of water storage (6 months) [41], thermocycling (10,000 cycles, approximately 1 year) [6, 45], and cyclic loading (120,000 cycles, approximately 1 year) [42]. Water storage at 37 °C induces hydrolytic degradation of both the resin material and the adhesive interface [41], whereas thermocycling and cyclic loading generate thermomechanical stresses at the crown-cement-tooth interface especially at the cervical margin, which is widely considered the most vulnerable region of bonded restorations [16, 46]. Also, the negative hydrolytic effect of water on the resin materials used for crown fabrication and cementation might result in a decrease in thermal stability and polymer plasticization of both restoration and luting materials due to possible water sorption and swelling stresses [16, 46]. Marginal gap results of the present study were higher than marginal gap results of other literatures [27, 28, 46]. This could be explained due to the use of natural teeth instead of standardized dies, the specific composition of the material used for crowns fabrication, and the measurement technique used.

### Study limitations

1. Include using one 3D printing material for crowns fabrication.
2. limited number of cyclic loading.
3. Additionally, marginal adaptation was assessed only prior to cementation and following artificial aging; thus, the individual effects of cementation film thickness and artificial aging degradation could not be separated.

## Conclusions

Within the limitations of this in vitro study, the following conclusions can be drawn:

1. Finish line design and workflow type each significantly affected the marginal adaptation of 3D-printed definitive crowns.
2. Specimens prepared with a heavy chamfer finish line exhibited significantly lower marginal gaps compared to those with a conservative chamfer design.
3. The direct digital workflow yielded superior marginal adaptation compared to the semi-digital workflow.
4. Although the differences were statistically significant, marginal gap values remained within the clinically acceptable threshold (120 μm) for most subgroups; the semi-digital conservative chamfer (CS) subgroup slightly exceeded this limit after the combined cementation and artificial aging.

## Data Availability

The data sets used and/or analyzed during the current study are available from the corresponding author upon reasonable request.

## Abbreviations

N: Newton
CAD/CAM: Computer aided design; computer aided manufacture
CEJ: Cemento-enamel junction
SPSS: Social Package for Statistical Science
SEM: Scanning electron microscope
H: Heavy chamfer
C: Conservative Chamfer
D: Direct digital intraoral scanning workflow
S: Semi-digital workflow

## References

[1] Duret F, Blouin JL, Duret B. CAD-CAM in dentistry. J Am Dent Assoc. 1988;117(6):715–20. 10.14219/jada.archive.1988.0096. 3058771

[2] Corbani K, Hardan L, Skienhe H, Özcan M, Alharbi N, Salameh Z. Effect of material thickness on the fracture resistance and failure pattern of 3D-printed composite crowns. Int J Comput Dent. 2020;23(3):225–33. https://pubmed.ncbi.nlm.nih.gov/32789310/. 32789310

[3] Watanabe H, Fellows C, An H. Digital Technologies for Restorative Dentistry. Dent Clin North Am. 2022;66(4):567–90. 10.1016/j.cden.2022.05.006. 36216447

[4] Kessler A, Hickel R, Reymus M. 3D Printing in Dentistry-State of the Art. Oper Dent. 2020;45(1):30–40. 10.2341/18-229-l. 31172871

[5] Tsirogiannis P, Reissmann DR, Heydecke G. Evaluation of the marginal fit of single-unit, complete-coverage ceramic restorations fabricated after digital and conventional impressions: A systematic review and meta-analysis. J Prosthet Dent. 2016;116(3):328–e335322. 10.1016/j.prosdent.2016.01.028. 27061627

[6] Alper ES, Simsek H, Buyuk SK, Guler C, Çadırcı M. Effect of different washing and post-curing protocols on the fracture resistance of 3D-printed pediatric crowns after thermal cycling. Sci Rep. 2026. 10.1038/s41598-026-54905-2. 42215587 PMC13454620

[7] Miyazaki T, Hotta Y. CAD/CAM systems available for the fabrication of crown and bridge restorations. Aust Dent J. 2011;56(Suppl 1):97–106. 10.1111/j.1834-7819.2010.01300.x. 21564120

[8] Abdel-Azim T, Rogers K, Elathamna E, Zandinejad A, Metz M, Morton D. Comparison of the marginal fit of lithium disilicate crowns fabricated with CAD/CAM technology by using conventional impressions and two intraoral digital scanners. J Prosthet Dent. 2015;114(4):554–9. 10.1016/j.prosdent.2015.04.001. 26100929

[9] Ahrberg D, Lauer HC, Ahrberg M, Weigl P. Evaluation of fit and efficiency of CAD/CAM fabricated all-ceramic restorations based on direct and indirect digitalization: a double-blinded, randomized clinical trial. Clin Oral Investig. 2016;20(2):291–300. 10.1007/s00784-015-1504-6. 26070435

[10] An S, Kim S, Choi H, Lee JH, Moon HS. Evaluating the marginal fit of zirconia copings with digital impressions with an intraoral digital scanner. J Prosthet Dent. 2014;112(5):1171–5. 10.1016/j.prosdent.2013.12.024. 24951386

[11] Anadioti E, Aquilino SA, Gratton DG, Holloway JA, Denry I, Thomas GW, Qian F. 3D and 2D marginal fit of pressed and CAD/CAM lithium disilicate crowns made from digital and conventional impressions. J Prosthodont. 2014;23(8):610–7. 10.1111/jopr.12180. 24995593

[12] Strub JR, Rekow ED, Witkowski S. Computer-aided design and fabrication of dental restorations: current systems and future possibilities. J Am Dent Assoc. 2006;137(9):1289–96. 10.14219/jada.archive.2006.0389. 16946436

[13] Chochlidakis KM, Papaspyridakos P, Geminiani A, Chen CJ, Feng IJ, Ercoli C. Digital versus conventional impressions for fixed prosthodontics: A systematic review and meta-analysis. J Prosthet Dent. 2016;116(2):184–e190112. 10.1016/j.prosdent.2015.12.017. 26946916

[14] Papadiochou S, Pissiotis AL. Marginal adaptation and CAD-CAM technology: A systematic review of restorative material and fabrication techniques. J Prosthet Dent. 2018;119(4):545–51. 10.1016/j.prosdent.2017.07.001. 28967399

[15] McLean JW, von Fraunhofer JA. The estimation of cement film thickness by an in vivo technique. Br Dent J. 1971;131(3):107–11. 5283545 10.1038/sj.bdj.4802708

[16] Rossetti PH, do Valle AL, de Carvalho RM, De Goes MF, Pegoraro LF. Correlation between margin fit and microleakage in complete crowns cemented with three luting agents. J Appl Oral Sci. 2008;16(1):64–9. 10.1590/s1678-77572008000100013. 19089292 PMC4327283

[17] Holmes JR, Bayne SC, Holland GA, Sulik WD. Considerations in measurement of marginal fit. J Prosthet Dent. 1989;62(4):405–8. 10.1016/0022-3913(89)90170-4. 2685240

[18] Li M, Ma B, Zhou Z, Liu W. Influence of impression method and shoulder design on the marginal adaptation of CAD/CAM nanoceramic resin onlay restorations. Heliyon. 2024;10(16):e35915. 10.1016/j.heliyon.2024.e35915. 39224323 PMC11367026

[19] Deranek K, Siegel SC, Golberg MB, Valdivieso AF. Tooth Preparation Assessment Criteria for All-Ceramic CAD/CAM Posterior Crowns: An Evidence Map. J Dent Educ. 2025;89(10):1464–78. 10.1002/jdd.13849. 39988901

[20] Shely A, Nissan J, Lugassy D, Rosner O, Zenziper E, Egbaria T, Ben-Izhack G. Three Self-Adhesive Resin Cements and Their Influence on the Marginal Adaptation of Zirconia-Reinforced Lithium Silicate Single Crowns: An In Vitro Scanning Electron Microscope Evaluation. J Clin Med. 2024;13(11). 10.3390/jcm13113330. PMC11173126 38893040

[21] Nasir MQ, Kadhim AJ. Marginal adaptation of different monolithic zirconia crowns with horizontal and vertical finish lines: A comparative in vitro study. J Dent Res Dent Clin Dent Prospects. 2023;17(4):235–41. 10.34172/joddd.2023.40589. 38584994 PMC10998165

[22] Singh R, Mistry G, Kini A, Ansari R, Kailaje V, Kapoor S. Accuracy and Clinical Performance of Intraoral Scanners Compared to Conventional and Extraoral Impressions. Umbrella Rev Cureus. 2025;17(9):e93202. 10.7759/cureus.93202. PMC12553488 41141051

[23] Husein HA, Morad ML, Kanout S. Accuracy of Conventional and Digital Methods of Obtaining Full-Arch Dental Impression (In Vitro Study). Cureus. 2022;14(9):e29055. 10.7759/cureus.29055. 36249650 PMC9554360

[24] Chee WW, Donovan TE. Polyvinyl siloxane impression materials: a review of properties and techniques. J Prosthet Dent. 1992;68(5):728–32. 10.1016/0022-3913(92)90192-d. 1432791

[25] Almeida e Silva JS, Erdelt K, Edelhoff D, Araújo É, Stimmelmayr M, Vieira LC, Güth JF. Marginal and internal fit of four-unit zirconia fixed dental prostheses based on digital and conventional impression techniques. Clin Oral Investig. 2014;18(2):515–23. 10.1007/s00784-013-0987-2. 23716064

[26] Topdagi B, Guler CC, Sultanoglu EG. Marginal and internal fit accuracy of single-crown restorations: the impact of digital and conventional impression techniques. BMC Oral Health. 2025;26(1):79. 10.1186/s12903-025-07491-0. 41361412 PMC12802305

[27] Hassan RM, Ibrahim Y, Abo ERG, Azer AS. Verification of extraoral versus intraoral scanning techniques: fit accuracy implications for 3D printed and milled zirconia crowns (An in-vitro study). BMC Oral Health. 2026;26(1):221. 10.1186/s12903-025-07577-9. 41545973 PMC12866500

[28] Abdulkareem MA, Al-Shamma AMW. Marginal adaptation and fracture resistance of 3D-printed and CAD/CAM-milled definitive resin matrix ceramic crowns. Int J Comput Dent. 2024;27(4):355–63. 10.3290/j.ijcd.b4494301. 37823542

[29] Yadav P, Sharma V, Paliwal J, Meena KK, Madaan R, Gurjar B. An In Vitro Comparison of Zirconia and Hybrid Ceramic Crowns With Heavy Chamfer and Shoulder Finish Lines. Cureus. 2023;15(1):e33940. 10.7759/cureus.33940. 36819334 PMC9937780

[30] Skjold A, Schriwer C, Øilo M. Effect of margin design on fracture load of zirconia crowns. Eur J Oral Sci. 2019;127(1):89–96. 10.1111/eos.12593. 30467907 PMC6587860

[31] Shillingburg HT, Hobo S, Whitsett LD, Jacobi R, Brackett SE. Fundamentals of fixed prosthodontics. Volume 194., IL, USA: Quintessence Publishing Company Chicago; 1997.

[32] Nedelcu R, Olsson P, Nyström I, Thor A. Finish line distinctness and accuracy in 7 intraoral scanners versus conventional impression: an in vitro descriptive comparison. BMC Oral Health. 2018;18(1):27. 10.1186/s12903-018-0489-3. 29471825 PMC5824445

[33] Park Y, Kim JH, Park JK, Son SA. Scanning accuracy of an intraoral scanner according to different inlay preparation designs. BMC Oral Health. 2023;23(1):515. 10.1186/s12903-023-03233-2. 37488581 PMC10367335

[34] Bonfanti-Gris M, Pradies G, Moron-Conejo B, Gil A, Martinez-Rus F. Vertical Versus Horizontal Finishing Lines for Dental Preparations: A Systematic Review With Meta-Analysis. J Esthet Restor Dent. 2025;37(3):707–26. 10.1111/jerd.13360. 39620436 PMC12076104

[35] Mümtaz G, Koyuncu B. Marginal and internal fit of 3D printed permanent crowns: effect of CAD/CAM software, preparation type and resin material. BMC Oral Health. 2026. 10.1186/s12903-026-08948-6. 42316147 PMC13520446

[36] Faul F, Erdfelder E, Lang AG, Buchner A. G*Power 3: a flexible statistical power analysis program for the social, behavioral, and biomedical sciences. Behav Res Methods. 2007;39(2):175–91. 10.3758/bf03193146. 17695343

[37] Sandhu SV, Tiwari R, Bhullar RK, Bansal H, Bhandari R, Kakkar T, Bhusri R. Sterilization of extracted human teeth: A comparative analysis. J Oral Biol Craniofac Res. 2012;2(3):170–5. 10.1016/j.jobcr.2012.09.002. 25737861 PMC3942122

[38] Linhares LA, Pottmaier LF, Lopes GC. Fracture resistance of veneers in premolars. Eur J Dent. 2018;12(2):191–8. 10.4103/ejd.ejd_349_17. 29988214 PMC6004806

[39] Elsayed MS, El-Kouedi AY, Shokry TE. Effect of aging on the marginal fit of milled and printed zirconia crowns: an in-vitro study. BMC Oral Health. 2025;25(1):221. 10.1186/s12903-025-05542-0. 39934766 PMC11817762

[40] Hammamy M, Rueda SR, Pio A, Rizzante FAP, Lawson NC. Effect of Air Particle Abrasion and Primers on Bond Strength to 3D-Printed Crown Materials. Mater (Basel). 2025;18(2). 10.3390/ma18020265. PMC11766915 39859736

[41] De Munck J, Van Landuyt K, Peumans M, Poitevin A, Lambrechts P, Braem M, Van Meerbeek B. A critical review of the durability of adhesion to tooth tissue: methods and results. J Dent Res. 2005;84(2):118–32. 10.1177/154405910508400204. 15668328

[42] Awad YA, Dawood L, El-Anwar M, Attia A. Fracture resistance of translucent zirconia overlay restoration with different preparation designs. BMC Oral Health. 2026;26(1):193. 10.1186/s12903-025-07456-3. 41527089 PMC12853914

[43] Attia A, Kern M. Influence of cyclic loading and luting agents on the fracture load of two all-ceramic crown systems. J Prosthet Dent. 2004;92(6):551–6. 10.1016/j.prosdent.2004.09.002. 15583561

[44] Attia A, Abdelaziz KM, Freitag S, Kern M. Fracture load of composite resin and feldspathic all-ceramic CAD/CAM crowns. J Prosthet Dent. 2006;95(2):117–23. 10.1016/j.prosdent.2005.11.014. 16473085

[45] Gale MS, Darvell BW. Thermal cycling procedures for laboratory testing of dental restorations. J Dent. 1999;27(2):89–99. 10.1016/s0300-5712(98)00037-2. 10071465

[46] Tartuk BK, Akın Tartuk G. Impact of Thermomechanical Aging on Marginal Fit and Fracture Resistance of CAD/CAM Endocrowns Fabricated from Different Materials. Polym (Basel). 2026;18(1). 10.3390/polym18010143. PMC12787759 41516926

